# Application of Pressure to Uterus in Cases of Lingering Labour

**Published:** 1871-03

**Authors:** 


### APPLICATION OF PRESSURE TO THE UTERUS IN CASES OF LINGERING LABOUR.

Von Ritgen, in 1856, suggested the employment of external pressure on the uterus as an adjuvant in cases of powerless labour, and the suggestion was put into practice by Kristellar, in 1867, and he published a number of cases in which he had advantageously resorted to it.

Dr. W. S. Playfair in an instructive article (*Lancet*, Oct. 1, 1870,) observes "It is certain the advantages to be derived from external pressure are not yet widely known or recognized; and as I have now received very material assistance from it in many cases of lingering and powerless labour, I believe it may not be without interest to state briefly the result of my experience on this point, especially as I do not know of any published cases in this country in which its use has been described.

"The class of cases in which external pressure is likely to prove serviceable is of very frequent occurrence, viz., in which the presentation is natural, and the pelvis roomy, but in which delivery is retarded simply from deficiency or absence of uterine contraction. These are the cases in which resort to the forceps is so often essential, in which the head has passed well into the pelvis, possibly descended as low as the perineum, and in which apparently but one or two good pains are required to complete the delivery.

"Firm pressure, applied under such circumstances, may act in two ways: First and most commonly, it may merely stimulate the sluggish uterus to increased exertion, just as firm pressure after delivery will cause a relaxed uterus to contract. In this way, pains that are feeble and ineffective may be rendered strong and useful, and a natural termination may result when artificial assistance might otherwise be required. I have of late been frequently in the habit of thus stimulating the uterus, and I feel certain that I have in many instances greatly shortened the progress of a labour that threatened to be long and tedious. It is, indeed, often curious to observe how rapidly the pains increase in force and duration; under the stimulation of gentle and steady pressure at the commencement of each pain." * * *

"I need hardly add, by way of caution, that gentle but firm pressure in a proper direction is to be used and that all rough handling of the uterus is to be avoided. The pressure can be most readily applied with the patient lying on her back, but this is by no means essential, and I have constantly used it in the ordinary position on the side, and without disturbing the patient. —*Am. Jour. of Med. Sciences.*

